# Allometric Scaling and Cell Ratios in Multi-Organ *in vitro* Models of Human Metabolism

**DOI:** 10.3389/fbioe.2014.00074

**Published:** 2014-12-17

**Authors:** Nadia Ucciferri, Tommaso Sbrana, Arti Ahluwalia

**Affiliations:** ^1^CNR Institute of Clinical Physiology, Pisa, Italy; ^2^Interdepartmental Research Center “E. Piaggio”, University of Pisa, Pisa, Italy

**Keywords:** allometry, *in vitro* models, organ-on-a-plate, metabolism, hepatocytes, endothelial cells

## Abstract

Intelligent *in vitro* models able to recapitulate the physiological interactions between tissues in the body have enormous potential as they enable detailed studies on specific two-way or higher order tissue communication. These models are the first step toward building an integrated picture of systemic metabolism and signaling in physiological or pathological conditions. However, the rational design of *in vitro* models of cell–cell or cell–tissue interaction is difficult as quite often cell culture experiments are driven by the device used, rather than by design considerations. Indeed, very little research has been carried out on *in vitro* models of metabolism connecting different cell or tissue types in a physiologically and metabolically relevant manner. Here, we analyze the physiological relationship between cells, cell metabolism, and exchange in the human body using allometric rules, downscaling them to an organ-on-a-plate device. In particular, in order to establish appropriate cell ratios in the system in a rational manner, two different allometric scaling models (cell number scaling model and metabolic and surface scaling model) are proposed and applied to a two compartment model of hepatic-vascular metabolic cross-talk. The theoretical scaling studies illustrate that the design and hence relevance of multi-organ models is principally determined by experimental constraints. Two experimentally feasible model configurations are then implemented in a multi-compartment organ-on-a-plate device. An analysis of the metabolic response of the two configurations demonstrates that their glucose and lipid balance is quite different, with only one of the two models recapitulating physiological-like homeostasis. In conclusion, not only do cross-talk and physical stimuli play an important role in *in vitro* models, but the numeric relationship between cells is also crucial to recreate *in vitro* interactions, which can be extrapolated to the *in vivo* reality.

## Introduction

Systems in which two or more organ or tissue models are connected or combined together have been proposed by several groups. These can generally be classified into three groups: co-cultures, microscaled multi-organ or body-on-a-chip, and milli-scaled multi-organ-on-a-plate. Most of the models reported in the literature involve co-cultures in which two cell types are seeded together (Turtzo et al., 2001; Zinchenko et al., 2006), or transwell cultures in which two cell types are separated by semi-permeable membranes (Lau et al., 2004). The more sophisticated models use fluidic systems or bioreactors to connect the different organ models together (Ouattara et al., 2011). A complex microfluidic bioreactor, μCCA, was developed by Viravaidya et al. (2003) combining three cell types in a multi-chamber device to test the toxicity of naphthalene. The system was designed using pharmacokinetic-pharmacodynamic scaling and is one of the few examples of cell culture system design based on mechanistic mathematical models. Such a device brings us a step closer to observing the systematic, whole-body response to drugs rather than the response of a single cell population, and is known as the “body-on-a-chip” approach (Esch et al., 2011).

Ahluwalia and co-workers first described the use of the principles of allometric scaling to establish design rules for *in vitro* models representing different physiological organ–organ interactions such as the lung–liver and intestinal–liver axes (Vozzi et al., 2009; Sbrana and Ahluwalia, 2012). Since then, some reports have speculated on the scaling of micro and milli-scaled multi-organ devices using allometry (Wikswo et al., 2013), suggesting that allometry breaks down at the microscale, and hence that multi-organ on a chip devices should perhaps not be “too small.” More recently, Moraes et al. (2013) propose a “metabolically supported functional scaling” approach, which is very similar to the metabolic scaling for the liver described in Sbrana and Ahluwalia (2012).

The multi-organ-on-a-chip concept has become quite popular, with several groups in the USA, Europe, and Japan attempting to design microscaled fluidic devices for drug and toxicity testing using more than one cell type (Zhang et al., 2009; Imura et al., 2012; Wagner et al., 2013). Still very much a niche tool limited to laboratories with microfabrication facilities and microfluidic pumps, they have nevertheless attracted considerable interest. Some of the practical limitations of these systems (bubbles, large surface areas, etc.) have been highlighted in Mattei et al. (2014), who also demonstrate that the constraint of maintaining low shear stress in microscaled fluidic devices may lead to severe glucose depletion, even if a sufficient oxygen supply is guaranteed by the presence of gas permeable (typically polydimethylsiloxane or PDMS) walls.

**Figure 1:**
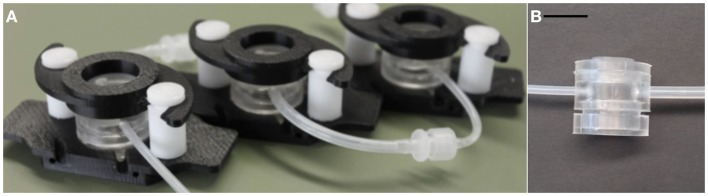
**(A)** Example of a MCmB circuit with three chambers, **(B)** A single MCmB module. Scale bar represents 10 mm.

On a different scale, connected cultures of hepatocytes, adipocytes, and endothelial cells in modular bioreactors (Figure 1) have been used to investigate the regulation of systemic metabolism *in vitro*. The milli-scaled MCmB system on-a-plate was designed using allometric scaling, focusing on glucose and lipid processing for their relevance to diabetes and metabolic disorders (Guzzardi et al., 2009). Successive investigations on the role of adipose tissue and the effects of over-nutrition in the systemic model were also reported (Vinci et al., 2012; Iori et al., 2012). Milli-scaled on-a-plate devices for multi-organ cultures are commercially available, but are not very widespread due to the necessity of fluidics (pumps) and the obligation of establishing a common culture medium.

The increasing number of papers on multi-cell and multi-organ systems nevertheless underlines the interest in this type of study, particularly as regards drug and toxicity testing and disease models. The underlying methodological assumption of these studies is that by combing different cell types together, it is possible to translate the results to the *in vivo* reality. However, most reports do not justify the cell types used, their ratios, or the particular culture conditions employed. On the other hand, in order to develop physiologically relevant models, it is important to properly design cell culture devices and use meaningful cell surface areas or cell ratios according to the type of experiment being conducted. In fact, one of the main conclusions of our studies on glucose and lipid metabolism was that to elicit meaningful responses, a fluidic connected culture system requires first cell numbers and ratios, which enable appropriate physiological-like interactions, and, second, flow rates that do not cause shear stress-related damage to cells and that allow adequate residence times in each compartment to enable cells to process metabolic signals, while permitting an adequate oxygen supply through convection (Mazzei et al., 2010).

Given the growing interest and the importance of appropriate scaling in multi-organ systems, in this paper allometric scaling models are proposed and applied to the study of metabolic regulation in a two compartment hepatic-endothelial model. We considered two models: one based on cell numbers (cell number scaling model, CNSM), and the second based on metabolic rates and cell surface areas (metabolic and surface scaling model, MSSM). Our aim was to use rational design considerations based on allometry to determine cell ratios in a multi-organ-on-a-plate device and to determine if and how differences in the initial conditions (i.e., cell numbers and ratios) can influence overall cell function and metabolic response.

For the sake of completeness, we first introduce the concept of allometric scaling and show how allometry can be used to evaluate the relationships between different tissues in order to ensure that the same relationships are conserved in a downscaled *in vitro* environment. After describing allometric scaling models, in the second part of this paper, we establish two experimentally feasible cell ratios based on the models and test them using a modular cell culture system specifically developed for reconstructing tissue and organ cross-talk *in vitro* (Vozzi et al., 2011).

## Allometric Scaling in *in vitro* Models

Allometry is the science of scaling and deals with changes in body size and relationships among different parameters and processes in all organisms as a function of body mass *M* (Lindstedt and Schaeffer, 2002; West and Brown, 2005). The basic allometric equation can be used to correlate physiological variables between organisms of different sizes.

(1) $$Y=a\times M^{b}$$

*Y* is the physiological parameter that has to be correlated with body mass (*M*), such as basal metabolic rate (BMR), heart rate, life span, etc. The constant *a* is a proportionality factor for the particular parameter, whereas *b* is the allometric exponent. *b* varies in magnitude and sign and has a specific value for each parameter according to how it scales with mass. Table 1 explains how *b* determines the manner in which physiological parameters change with mass.

**Table 1:** **The value of *b* determines how a physiological parameter varies with body mass**.

*b*	Significance	Example (value of *b*)
0	Parameter does not change with body mass	Bone density in mammals, cell radius
1	Parameter changes in direct proportion with body mass	Body volume, cell number
0 < *b* < 1	Parameter increases at a slower rate than body mass	Metabolic rate (3/4), blood flow rate (3/4), external surface area (2/3), life span (1/4)
>1	Parameter increases at a faster rate than body mass	Bone mass (4/3)
<0	Parameter decreases with body mass	Almost all frequencies or rates, e.g., cardiac frequency, respiratory frequency (−1/4)

The allometric approach can be used to evaluate the relationships between different tissues in order to ensure that the same relationships are conserved in a downscaled *in vitro* environment. In this paper, the approach is applied to scale a hepatic-endothelial model in the MCmB, a Multi-compartment modular Bioreactor system, which enables different chambers to be connected together in a fluidic circuit (Mazzei et al., 2010). It consists of interconnected chambers with dimensions similar to that of a 24-plate well, each one designed to house a specific tissue or organ (Figure 1). Higher order more complex models can thus be assembled by combining two or more cell types or tissues connected by the flow of a common medium and modules can simply be added or removed to alter the contribution of each organ in the model.

### Metabolic and surface scaling model

The liver is responsible for the uptake, conversion, and distribution of many of the nutrients entering the digestive tract and is also the main orchestrator of exogenous metabolism while vascular tissue is the conduit through which signals are relayed to distant organs. We therefore begin by connecting hepatocytes with endothelial cells before adding other cells or tissues to construct an *in vitro* model of biotransformation and distribution. Biotransformation is a metabolic process and depends on the metabolic efficiency of cells, whereas distribution is a surface mediated process. Therefore, the hepatocytes in the model are scaled with reference to basal metabolism whereas the endothelium is scaled using the surface area of the human vascular system as a starting point. As cells are usually plated in monolayers, the allometric design process begins by considering the metabolism of a two dimensional culture of human hepatocytes in a single module. Hepatocytes can be seeded on the bottom surface of the chamber (either on a slide or a scaffold) to represent the liver of a multi-organ *in vitro* model.

Standard parameters and their corresponding bibliographic references used to establish the experimental set-up in the MSSM through allometric scaling are summarized in Table 2. The liver generates 27% of the total BMR of a human, corresponding to 23.76 W. Then, assuming the total metabolic contribution of the liver is due principally to its approximately 200 billion hepatocytes, the BMR per human hepatocyte is 119 pW. The surface area of the cell culture zone of a chamber is the same as that of a 24-plate well: 1.33 cm^2^. A confluent layer of hepatocytes has a density of about 2 × 10^5^ cells/cm^2^, which corresponds to 2.6 × 10^5^ cells in the chamber (Ferrini et al., 1998), therefore the equivalent BMR of the *in vitro* one chamber liver is 30 μW. Since we are using human hepatocytes in our model, we assume that their metabolic contribution remains unchanged (Porter, 2001), that is they contribute to 27% of the downscaled organism’s BMR. Thus, the total BMR of the system with 2.6 × 10^5^ hepatocytes is 111 μW (*BMR_in_*).

**Table 2:** **Summary of data used for allometric scaling of liver (Bean, 1926; Durnin, 2002; Sohlenius-Sternbeck, 2006)**.

Human data	Value
Body mass, *M*_ma_*n*	70 kg
Basal metabolic rate (BMR)	88 W
Liver mass	1.8 kg
Liver contribution to BMR	27%
No. of hepatocytes	2 × 10^11^

Allometric equations can be used in order to find the equivalent body mass (*M_in_*) of our *in vitro* system. Equations 2 and 3 summarize the steps required to estimate *M_in_* starting from the BMR and the mass of the human body (*BMR_man_* and *M_man_*, respectively).

(2) $${\mathrm{BMR}}_{\mathrm{man}}=a_{\mathrm{BMR}}\times {M_{\mathrm{man}}}^{3∕4}$$

(3) $${\mathrm{BMR}}_{\mathrm{in}}=a_{\mathrm{BMR}}\times M_{\mathrm{in}}^{3∕4}$$

*M_in_* in this case is 1 mg. Note that this value depends very much on the number and source of hepatocytes employed. *M_in_* can be up to 5 mg in three-dimensional scaffolds and more in tissue slices. Having established this parameter, the allometric approach can be used in order to find a suitable surface area to simulate the vascular endothelial exchange area. The allometric equation that links vascular surface area (*S*) to body mass (*M*) of a mammal is (Dawson, 2003):

(4) $$S=a\times M^{11∕12}$$

Note that the vascular bed is a space filling structure; therefore, the capillary area does not follow the 2/3 scaling law, which only holds for external surface areas. Given that capillary bed surface of a standard man in resting conditions is 500 m^2^ (Kamiya et al., 1987), we can estimate the constant *a* from Eq. 4 and then find a suitable surface area to simulate the endothelium in an *in vitro* experiment.

(5) $$S=a\times {\left(1\;\text{mg}\right)}^{11∕12}$$

The surface of a chamber that represents the endothelium should be 0.33 cm^2^. This is about a quarter of the cell culture area of an MCmB module. Iterating through Eqs 2–5, it is easy to show that if one chamber is used to simulate the exchange surface area of the endothelium, about three to four modules have to be used to represent the liver (less if hepatocyte density is increased by seeding on a scaffold). Note that the relationship is not linear due to the exponent based scaling laws used.

### Cell number scaling model

An alternative model is described to simulate cross-talk between endothelial and hepatic tissue. In tissues, cell numbers play an important role in physiological functions. For example, cell number is an important parameter in order to characterize drug filtration or absorption rates. Thus, if the aim is to study drug passage from one organ to another by simulating tissues as monolayers in several bioreactor chambers connected in series or in parallel, the cell numbers in each culture are a key point in the experimental set up. An allometric model based on cell numbers could represent a valid alternative to the MSS model in order to simulate cross-talk between tissues. Vascular endothelial tissue represents 6.28% of total body weight (NASA, 1995), while liver mass is equal to 2.6% of human body mass.

Given that hepatocytes represent 60% of liver mass (Sohlenius-Sternbeck, 2006), Eq. 6 enables calculation of the ratio (*r*) between the body’s endothelial mass (*M_endo_*) and hepatocyte mass (*M_hep_*):

(6) $$r=\frac{M_{\mathrm{endo}}}{M_{\mathrm{hep}}}\approx 4$$

“*r*” represents physiological ratio between endothelial and hepatocyte mass in the body. If we suppose that both cells have the same average mass, as suggested by the data on endothelium thickness (0.3 μm) and diameter (Pries et al., 2000) and hepatocyte size (Uhal and Roehrig, 1982), then in order to maintain this correlation in an *in vitro* experiment, the endothelial number has to be around four times the hepatocyte cell number. This assumption is also supported by Kozlowski et al. (2010) who demonstrate that cell mass is size invariant in mammals.

Since endothelial cells in culture are almost three times larger in diameter than hepatocytes *in vitro* (Haas and Duling, 1997; Pries et al., 2000; Vizzotto et al., 2001), the value of *r* calculated results in a 36:1 ratio of endothelial:hepatic bioreactors. Assembling 36 chambers with endothelial cells would not only lead to large fluid volumes, which may increase metabolite dilution, but is also impractical using the MCmB modules. Therefore, as an alternative to scaling down whole-body cell numbers, we focused on the ratio between endothelial cells and hepatocytes in the visceral abdominal region. This region is the first to receive nutrients after intestinal processing and is relevant to short term metabolism of glucose and lipids (Kuo and Ma, 2001; Iori et al., 2012).

The abdomen represents 3.08% of body mass – most of this is liver (Bean, 1926). Assuming that there is a uniform distribution of vascular tissue in the body, we can evaluate abdominal vascular tissue mass (*M_avt_*) using the following equation:

(7) $$M_{\mathrm{avt}}=0.0308\times \left(M_{\mathrm{man}}\times 0.0628\right)$$

Then *r_a_*_,_ the physiological ratio between endothelial and hepatocyte mass in the abdomen is:

(8) $$r_{a}=\frac{M_{\mathrm{avt}}}{M_{\mathrm{hep}}}=0.08\approx 0.1$$

Again, to maintain this correlation in an *in vitro* experiment, the number of endothelial cells has to be around 1/10 of the hepatocyte number, but in this case the value of *r_a_* calculated conveniently results in a 1:1 ratio of bioreactors when employing monolayer monocultures.

### Whole body and abdominal MSSM and CNSM

For the sake of comparison, we show how the two models differ in surface area and cell number for both whole body and abdominal compartments in Table 3. The table underlines how the choice of models is inevitably conditioned by experimental and practical constraints related to the cell culture system being employed.

**Table 3:** **Comparison between the metabolic and cell number scaling models for the whole body and the abdominal region using monolayer cultures in the MCmB system**. The table underlines that the choice of model is strongly conditioned by experimental constraints.

	MSSM		CNSM	
	Hepatocytes	Endothelial area	Hepatocytes	Endothelial cells
Whole Body	1040000 cells (4 chambers)	1.33 cm^2^ (1 chamber)	260000cells (1 chamber)	942000 cells (36 chambers)
Abdominal	15 × 10^6^ cells (57 chambers)	1.33 cm^2^ (1 chamber)	260000 cells (1 chamber)	26000 cells (1 chamber)

For example, from Table 3 it is clear that the abdominal MSSM would necessitate either 57 hepatic chambers or several high density 3D hepatocyte cultures to implement. Given the very high hepatic cell number in the abdominal model, liver metabolism would likely predominate with respect to any endothelial related uptake or release of metabolites. On the other hand, the whole-body MSSM can be assembled simply by connecting together four 24-well-sized modular chambers seeded with monolayers of hepatocytes and one chamber seeded with endothelial cells. Vice-versa, as demonstrated, the CNSM for the whole body requires tens of endothelial chambers, while the abdominal model with its 1:1 hepatocyte to endothelial module ratio, is simple to set-up.

The main difference between the two models is that the MSSM considers whole-body metabolism and nutrient distribution and gives more weight to hepatic metabolism than the CNSM, since *b* = 3/4 for metabolism and almost 1 (11/12) for vascular surface area (see Table 1). On the other hand, the CNSM simply considers the mass ratios, independent of cell function. As the liver is the predominant metabolic organ in the visceral region, were we to evaluate the chamber ratios using MSSM for just the abdomen, we would require a large number of hepatocytes with respect to endothelial surface area.

In the following section, we describe the hepatic-endothelial experiments based on the two practicable allometric models (respectively the whole-body MSSM and the abdominal CNSM) in the MCmB system and show how changing cell numbers and ratios can condition experimental outcomes.

## Experimental

### Materials

Eagle’s Minimum Essential Medium (EMEM), Penicillin/Streptomycin/Amphotericin B, l-Glutamine was purchased from Lonza Bioscience (Basel, Switzerland). Fetal bovine serum (FBS) was purchased from PAA (Pasching, Austria); all other reagents were purchased from Sigma-Aldrich (St. Louis, MI, USA) unless otherwise specified.

### Endothelial extraction and culture

Human umbilical vein endothelial cells (HUVEC) were extracted from donor umbilical cords using collagenase solution treatment following the protocol from Baudin et al. (2007). A single donated umbilical cord was sufficient for all the experiments. They were donated through written and informed consent and the study was approved by the local ethical committee.

The medium for culturing endothelial cells was EMEM (with 5 mM glucose) supplemented with 10% FBS, 1% Penicillin/Streptomycin/Amphotericin B, 2 mM L-Glutamine; 1% non-essential amino acids 100×; 1% MEM vitamins solution; 10 μg/mL endothelial cell growth supplement (ECGS), 10 ng/mL human epidermal growth factor (hEGF); 3 ng/mL basic fibroblast growth factor (bFGF); 1 μg/mL hydrocortisone, 10 μg/mL heparin sodium salt and (henceforth named the “common” medium). The endothelial cells were routinely trypsinized and used between the third and eighth cell passages.

Twenty-four hours before the experiment, the cells were seeded at a confluent concentration of 20,000 cells/cm^2^ on 13 mm diameter plastic slides (Nunc, Denmark) precoated with 1% gelatin.

### Hepatocyte culture

The human hepatoma-derived cell line C3A (ATCC Culture, USA) was used in the experiments as this cell line retains most endogenous metabolic functions of human hepatocytes. Cells were cultured in EMEM with 10% FBS, 1% Penicillin/Streptomycin/Amphotericin B, 1% l-Glutamine 200 mM; 1% non-essential amino acids 100×, and 1% MEM vitamins solution (named complete EMEM). The C3A hepatocytes were seeded at a near confluent density of 200,000 cells/cm^2^ on glass coverslips. Before seeding, the glass coverslip was coated with collagen: 200 μL of 0.2 mg/mL of type I collagen was pipetted and allowed to cross link for about 30 min at 37°C. Collagen was prepared by extracting acid-soluble collagen from rat tail tendons (Beken et al., 1998).

### Dynamic cell culture

**Figure 2:**
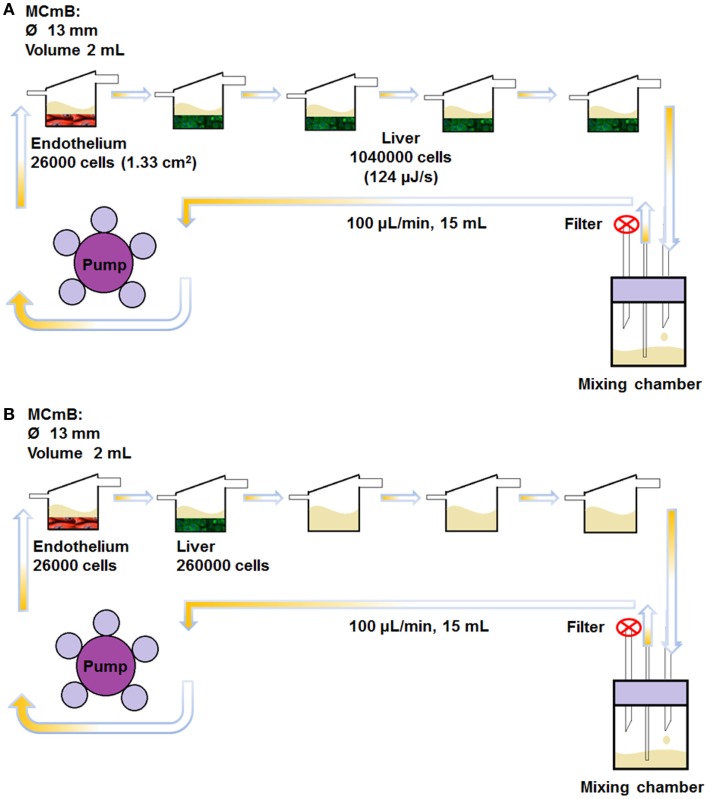
**The MCmB connection scheme for the whole-body MSSM (A) and the abdominal CNSM (B)**.

In this work, we used four (monolayer) hepatocyte chambers and one endothelial chamber to represent whole-body MSSM type scaling. The abdominal CNSM configuration was composed of one hepatocyte chamber and one endothelial chamber. All dynamic experiments were carried out in five MCmB modules connected in series as shown in Figure 2. For the MSSM, one endothelial cell coated slide was placed in the bottom of the first chamber and four hepatocyte slides were transferred to each of the other chambers of the system (Figure 2A). In the CNSM, one endothelial slide was placed in the bottom of the first chamber and one hepatocyte slide in the second chamber, the other three chambers were left empty of cells (Figure 2B). Each bottom part of the chamber was filled with 500 μL of common media in order to avoid drying of cells during assembly of the system. The rest of the common media (15 mL in total, necessary to fill the circuit and avoid nutrient depletion) was added to the mixing bottle. The circuits were closed and connected to a pump (Ismatech IPC-4, Zurich, Switzerland) and run at 100 μL/min in the incubator for 72 h.

As static controls, and to assess the effects of flow, the same number of coverslips were placed in a Petri dish with 15 mL of common medium and incubated for the same experimental time. Note that the volume of media was kept the same in all experiments, to reduce the number of variables in the system.

### Assays

At the end of the experiment, cells were analyzed for viability, morphology, and cytochrome activity. Viability was assessed by the CellTiter-Blue^^®^^ Cell Viability Assay (Promega) and compared with internal controls in multiwell plates. After viability evaluation, hepatocytes were also analyzed for Cytochrome P450 enzyme activity by VIVID^®^ CYP3A4 Red Screening Kit (Invitrogen Ltd, Paisley, UK); the increase in fluorescence emission after substrate addition is a measure of CYP3A4 activity. Cell morphology was assessed using phase contrast microscopy. The medium was analyzed for glucose (Glucose Test Kit, Megazyme International Ltd, Ireland), urea (Urea Kit, Sigma-Aldrich), lactate, glycerol, triglycerides (TG), and free fatty acids (FFA) (Lactate, Free Glycerol Assay kit, Triglyceride quantification kit, and Free Fatty Acid Quantification Kit were all from Biovision Inc., Milpitas, CA, USA). Human albumin concentration was determined by an ELISA immunochemical assay (Bethyl Laboratories, Montgomery, USA).

### Data analysis

Results were calculated from at least three different experiments and expressed as means ± SD of the mean. Data were analyzed using the Student’s *t*-test. Statistical significance was set at *p* < 0.05 (indicated with *) and high significance was set at *p* < 0.01 (marked with **).

## Results

A number of metabolic and functional assays were performed to assess the differences between the two cell ratios used in the experiments. All results are expressed as the difference between measured values and fresh media values. When the measured value was higher than that of fresh media, we denote this as release while measured values lower than fresh medium are denoted as uptake.

### Viability and P450 activity

**Figure 3:**
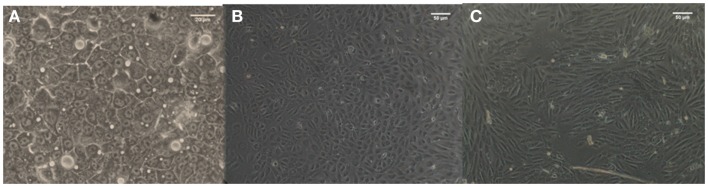
**Morphology of C3A cells (A) and HUVEC cells in static (B) and dynamic (C) conditions**.

Viability and P450 activity were evaluated on each cell coated coverslip. Both cell types in both experimental models showed high viability, with very little scatter. The vitality values were within 15% of the initial vitality, indicating that cell numbers remained essentially constant during the experiments. There was no difference in hepatocyte P450 activity in all conditions (data not shown). Furthermore, hepatocyte morphology was similar to that of our static controls (Figure 3A), with endothelial cells conserving a cobblestone-like morphology in static experiments and a slightly more elongated form in dynamic experiments (Figures 3B,C).

### Carbohydrate metabolism

**Figure 4:**
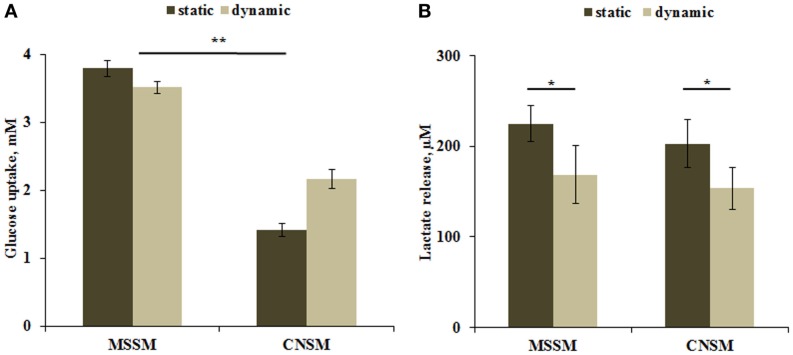
**(A)** Glucose uptake and **(B)** Lactate release in the two allometric model configurations. Static samples were the same type and number of cells cultivated in a petridish. Mean ± SD, *p < 0.05, **p < 0.01, *n* = 3.

Carbohydrates are the primary source of energy and metabolic intermediates. Glucose is the main carbohydrate used by the cell in both aerobic and anaerobic respiration. Hepatocytes are able to store or release glucose through glycogen synthesis or glycolysis pathways. Glucose dosing in the media showed a higher uptake in the MSSM configuration. In both static and dynamic conditions, the higher consumption of glucose can be attributed to the higher number of cells with respect to the CNSM model (approximately threefold more). Significant (*p* < 0.01) differences were observed between the two models in static conditions (Figure 4A). Lactate levels are a function of both glycolysis and gluconeogenesis in hepatocytes (Phillips et al., 1995), and are known to be down regulated by circulating (*in vivo*) or media FFAs (*in vitro*) (Morand et al., 1993). A significant (*p* < 0.05) decrease of lactate release in dynamic conditions was observed. No difference was found between the two models even when relating lactate release with glucose uptake: similar concentrations of lactate were found in both configurations despite the lower uptake of glucose in the CNSM (Figure 4B).

### Fat metabolism

The liver has a major role in the regulation both of glucose metabolism and fat metabolism. It can either oxidize or synthesize fatty acids and TG directing them to energy production or to storage. FFA and glycerol provided by ingestion or by drawing on TG are used as substrates for ATP production.

**Figure 5:**
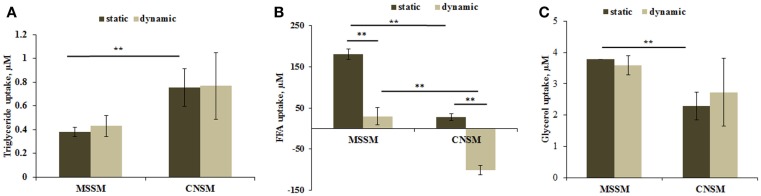
**(A)** Triglyceride uptake, **(B)** FFA uptake, and **(C)** glycerol uptake from cells in the two allometric model configurations. Static samples were the same type and number of cells cultivated in a petridish. Mean ± SD, *p < 0.05, **p < 0.01, *n* = 3.

Triglyceride dosing showed that cells in the CNSM configuration took up about twice as much triglyceride content as the MSSM even though the total amount of cells present in the system was four times less. No significant change was observed between the static and dynamic set up in both models (Figure 5A).

Free fatty acids data showed a variable trend between the two configurations. In the MSSM configuration, high FFA uptake was observed for the static set up with a lower uptake in the dynamic set up. In the CNSM, the slight uptake in static conditions was contrasted by a highly significant release in the dynamic configuration (Figure 5B). This inversion of the trend is likely caused by endothelial cells. In previous studies on endothelial cells, we observed high levels of FFA release in dynamic conditions compared with static experiments, whereas hepatocytes take up FFA in both static and dynamic conditions (Vinci et al., 2010). Finally, the same level of glycerol was taken up by cells both static and dynamic states in the two configurations, with an overall lower uptake in the CNSM with respect to MSSM (Figure 5C).

### Functional markers

**Figure 6:**
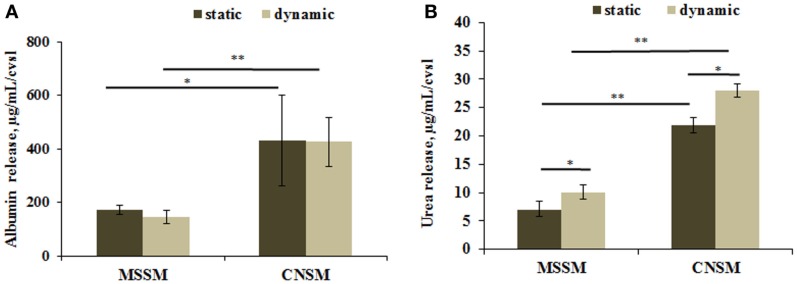
**(A)** Albumin release and **(B)** urea release from cells in the two allometric model configurations. Static samples were the same type and number of cells cultivated in a petridish. Data reported as concentration per coverslip; mean ± SD, *p < 0.05 and **p < 0.01, *n* = 3.

Healthy hepatocytes are able to perform carbohydrate, lipid, and xenobiotic metabolism, they also synthesize several molecules including albumin, and convert ammonia into urea. High levels of albumin and urea are considered to be positive indicators of hepatic function. A significant increase in albumin release was detected in the CNSM configuration where, despite the lower number of C3A, the cells release twice more albumin than in the MSSM. The content of albumin in each configuration was similar in static and dynamic conditions (Figure 6A). Urea release had the same trend as albumin, as shown in Figure 6B. Significantly higher amounts of urea were released from cells in the CNSM configuration (*p* < 0.01) and even more in the dynamic set up (*p* < 0.05).

## Discussion

In this study, two allometric models were used to scale metabolic interactions in the hepatic-endothelial axis in order to define rational design criteria for establishing cell ratios in a multi-organ-on-a-plate system. The CNSM was based on the scaling of cell numbers, while the MSSM was derived from considerations on hepatic metabolism and endothelial exchange. Using human data as a starting point, the results of allometric downscaling demonstrate that the cell ratios are considerably different for the two models and, as expected, depend on whether the whole body or core hepatic-endothelial axis is considered. In addition, the results in Table 3 show that only two models are feasible in the multi-organ-on-a-plate system under investigation.

The two practicable models were then evaluated experimentally to identify which of the two cell ratios better represents a more physiological state in terms of overall cell function and nutrient metabolism. The two systems examined represent, respectively, an allometrically scaled metabolic and surface area based scaling model of the whole-body relationship between endothelial cells and hepatocytes, and a model which albeit allometric, simply reflects the proportion of hepatocytes to endothelial cells in the abdomen.

Cell viability, P450 activity, and cell morphology were similar in both models, indicating that differences in cell ratios did not alter intrinsic cell function but rather their metabolic cross-talk. The analysis was thus centered on the balance between glucose and lipid concentrations in the media and the capacity of the system to recapitulate a physiological energy balance.

Overall, cells in the MSSM took up more glucose from the medium (Figure 4A). However, if we consider the glucose uptake per cell, then cells in the CNSM dynamic set up consume more glucose per cell with respect to the MSSM, but were nonetheless able to maintain glucose levels closer to normoglycaemic (~3 mM). Similarly, higher amounts of lactate per cell were released in the CNSM, reflecting the increased glucose uptake, and reaching total lactate concentrations similar to the MSSM configuration (Figure 4B).

The second most important mechanism for energy production in humans is fat metabolism. Significantly (*p* < 0.01) higher triglyceride uptake was shown in the CNSM (Figure 5A), resulting in a media triglyceride concentration per cell in the dynamic set up of about eightfold less with respect to the concentration per cell in the MSSM. This suggests that cells in the CNSM were undergoing higher triglyceride turnover than in the MSSM, so indicating that in the CNSM cells were producing energy by lipid metabolism at a higher level than in the MSSM. The FFA uptake results are also interesting and highly significant (*p* < 0.01) (Figure 6B). Vinci et al. (2010) have shown that FFA uptake in hepatocytes does not differ between the static and dynamic set up; in contrast endothelial cells are highly sensitive to flow and the FFA turnover is inverted: the cells uptake FFA in static conditions and release it in high amounts under flow conditions. Keeping this in mind, the data in Figure 5B, can be interpreted as an additive effect with both a hepatic and endothelial contribution. In static conditions, the uptake of FFA is due to hepatocytes, which are present in higher numbers than endothelial cells in both models. In fact, FFA uptake is higher in the MSSM (sixfold more) than that in CNSM, which has fewer cells. In dynamic conditions where endothelial cells competed with hepatocytes in the direction of FFA movement, lower uptake was shown in the MSSM as number of endothelial cells releasing FFA cannot overpass the high uptake of hepatocytes. On the other hand, the FFA trend was inverted in the dynamic CNSM configuration, where high FFA release from endothelial cells overrode the hepatic uptake, resulting in a net release. This high secretion of FFA can be due to the higher metabolism of TG and suggests that this molecule was not only up taken but also metabolized. Overall, the CNSM equilibrates glucose intake with a high fatty acid turnover and utilization.

Glycerol data showed no difference between static and dynamic conditions (Figure 5C), as confirmed by our previous studies (Vinci et al., 2010). Although the MSSM uptake was slightly more than in the CNSM, it is likely that the glycerol balance is modulated by the high triglyceride metabolism in the CNSM, rather than a lack of glycerol movement.

Overall, the metabolic data show that the CNSM consumes more glucose per cell while maintaining glucose levels closer to “normoglycemic” and has a higher level of fat metabolism and corresponding metabolite turnover. These data were confirmed by the assays on hepatic function. In fact, albumin concentration per cell was twice as much in the CNSM with respect to the MSSM in both static and dynamic conditions, indicating that cell cross-talk rather than the convective flow was the major driving force for the difference in albumin production (Figure 6A). Urea production per cell was also higher in the CNSM then in the MSSM (Figure 6B), and significantly upregulated by flow in both models.

In summary, we show that changing the initial conditions in a given multi-organ set-up can dramatically alter the metabolic balance in the system. The reason for the difference is due to the experimental set up, which will differ from device to device, rather than the allometric considerations made *per se* when scaling cell numbers or hepatic metabolism and vascular exchange area. We also demonstrate that if designed and tested in a rigorous manner, properly chosen cell ratios can be used to model some features of human metabolism, as demonstrated for the CNSM. We should, however, underline that in the MSSM we started off assuming a metabolic *in vitro* model in the form of a hepatocyte monolayer. This kind of organization is poorly correlated with organs such as liver in which the three-dimensional hierarchical structure is intimately linked with its metabolic function (Madsen, 2012). Physiological barriers or tissues involved in transport and exchange such as the endothelium, on the other hand, are better correlated with a two-dimensional structure than a metabolic organ.

This study demonstrates therefore that *in vitro* models of multiple organ systems need to be carefully designed and scaled, within the limits of experimental and practical constraints, in order to correctly represent physiological conditions. It also raises questions about the physiological relevance of current emerging integrative models and the scarcity of rational scaling and design in many of the systems reported. Our data show that not only do cross-talk and physical stimuli such as flow play an important role within an *in vitro* model but the exact metabolic and numerical relationship between cells is also crucial to recreate an appropriate *in vivo* like environment in which cells can maintain a homeostatic balance. In fact, if inappropriate or random scaling relationships are employed, diseased states such as hyperplasia or other pathologies manifested by anomalous relationships between cell numbers or cell signaling, rather than physiological models, can be recreated *in vitro*.

Finally, whatever model is used, the reader should bear in mind that models are, by definition, simplified depictions of reality. In this context, experimental *in vitro* models are necessarily a simplification of the more complex living organism. A good scientist should be aware of the limitations and boundary conditions of such tests, being cautious about over-interpretation of results. In fact, how accurately the experiments recapitulate *in vivo* conditions depends on the study design as well as the questions being posed and the desired outcomes.

## Conclusion

Multi-organ models are the frontier of *in vitro* research. Our work was driven by the great interest in developing new models capable of recapitulating systemic and multiple pathway interaction, and clearly shows that changing the relationships between cells in a multi-organ or multi-tissue system can lead to results, which may not be physiologically relevant, and even worse, incorrect.

This study provides new insights into advanced and rational model design and is, as far as we know, the first investigation of different cell ratios and allometric relationships between cells in a multi-organ or multi-tissue *in vitro* system.

## Conflict of Interest Statement

The authors declare that the research was conducted in the absence of any commercial or financial relationships that could be construed as a potential conflict of interest.
